# Tanshinone IIA attenuates AOM/DSS-induced colorectal tumorigenesis in mice via inhibition of intestinal inflammation

**DOI:** 10.1080/13880209.2020.1865412

**Published:** 2021-02-03

**Authors:** Lijie Liu, Hanjing Gao, Tao Wen, Tao Gu, Shuang Zhang, Zhiyong Yuan

**Affiliations:** a Department of Radiation Oncology, Tianjin Medical University Cancer Institute and Hospital, Key Laboratory of Cancer Prevention and Therapy, Tianjin, China; b Department of Oncology, First Hospital of Qinhuangdao, Qinhuangdao, China; c Department of Radiation Oncology, Tianjin 4TH Centre Hospital, Tianjin, China; d Medical Research Center, Beijing Chao-Yang Hospital, Capital Medical University, Beijing, China; e Department of Cardiology, First Hospital of Qinhuangdao, Qinhuangdao, China

**Keywords:** Intestinal permeability, neutrophil, NF-κB pathway

## Abstract

**Context:**

Tanshinone IIA is a natural extract derived from a Chinese medicinal herb with multiple bioactivities; however, whether and how tanshinone IIA protects against colorectal cancer (CRC) are uncertain.

**Objective:**

We investigated the potential beneficial effects of tanshinone IIA in a colitis-associated colorectal tumorigenesis mouse model and its underlying mechanisms.

**Materials and methods:**

Male C57BL/6 mice were treated with azoxymethane (AOM) 10 mg/kg body weight and dextran sulphate sodium (2.5% DSS) to induce a colitis-associated cancer model. Tanshinone IIA (200 mg/kg body weight) was given to the mice intraperitoneally. After 12 weeks, all mice were sacrificed to measure tumour formation, intestinal permeability, neutrophil infiltration, and colonic inflammation. In addition, whether tanshinone IIA has inhibitory effects on neutrophil activation was determined through *in vitro* investigations.

**Results:**

We observed that tanshinone IIA significantly decreased tumour formation in AOM/DSS-treated mice compared to AOM/DSS-treated alone mice (0.266 ± 0.057 vs. 0.78 ± 0.153, *p* = 0.013). Tanshinone IIA also decreased intestinal permeability compared to that in AOM/DSS-treated alone mice (3.12 ± 0.369 vs. 5.06 ± 0.597, *p* = 0.034) and consequently reduced neutrophil infiltration of the colonic mucosa (53.25 ± 8.85 vs. 107.6 ± 13.09, *p* = 0.014) as well as intestinal inflammation in mice. Mechanistically, tanshinone IIA downregulated the NF-κB signalling pathway in the colonic tumours of AOM/DSS-treated mice. *In vitro* assays further validated that tanshinone IIA suppressed LPS-induced neutrophil activation.

**Conclusion:**

These data suggest that tanshinone IIA alleviates colorectal tumorigenesis through inhibition of intestinal inflammation. Tanshinone IIA may have a therapeutic potential for CRC in clinical practice.

## Introduction

Colorectal cancer (CRC) is one of most common cancers worldwide and a leading cause of cancer-related death (Siegel et al. 2019). The clinical treatment options for CRC include surgery, chemotherapy, radiotherapy, and targeted therapy. Despite current advances and improvements in the early screening and therapy of CRC, the overall survival rates of CRC patients are discouragingly low (Siegel et al. 2019). Many risk factors have been associated with the aetiology of CRC; of these, chronic intestinal inflammation is a major risk factor (Beaugerie et al. 2013; Johnson et al. 2013; Lutgens et al. 2013). Accumulating evidence has demonstrated that people with inflammatory bowel disease (IBD), including ulcerative colitis and Crohn’s disease, have a 5- to 10-fold increase in the risk of developing CRC (Deng et al. 2019). Persistent inflammation alters immune responses, promotes the release of pro-inflammatory cytokines and growth factors, and further facilitates cell proliferation and tumorigenesis (Malgorzata et al. 2013; West et al. 2015; Yahyapour et al. 2018; Wang et al. 2020). Therefore, anti-inflammatory strategies in the intestine may be an attractive way to prevent and treat CRC and are worthy of more investigation.

**Figure 1.**
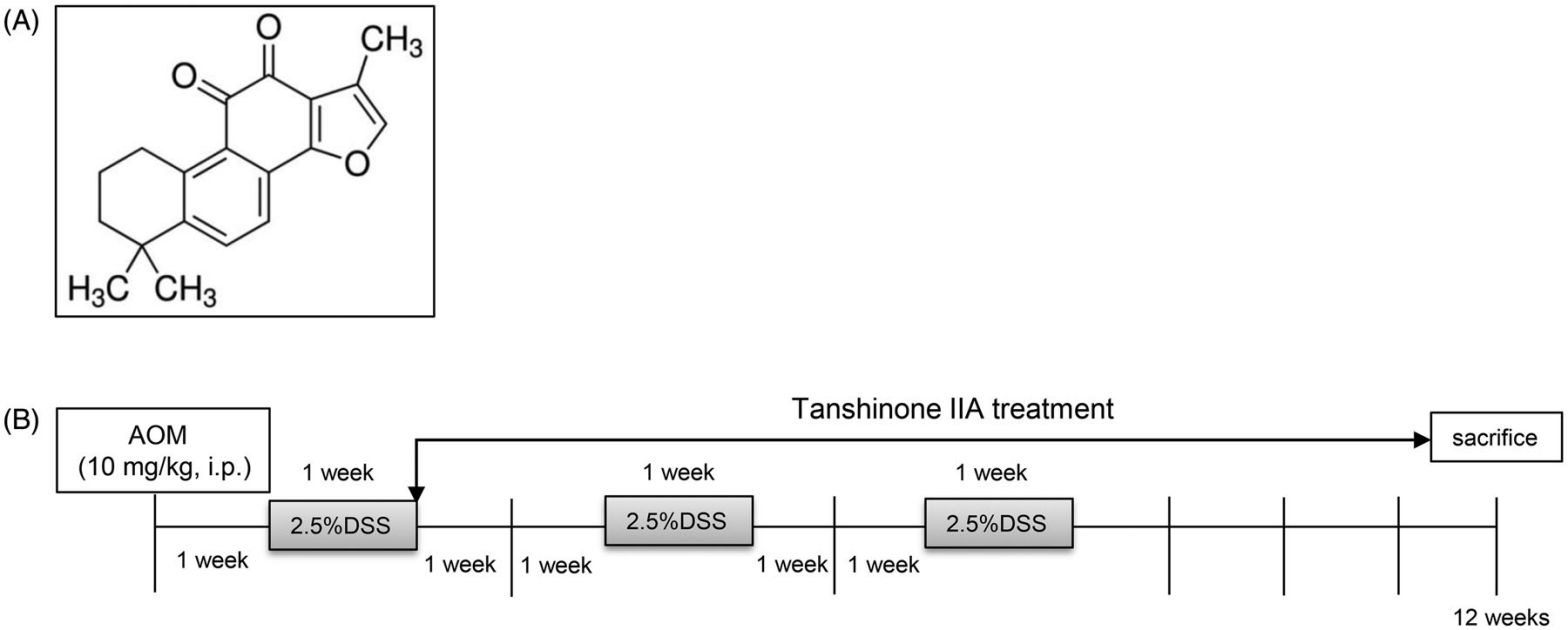
Experimental scheme. (A) Chemical structure of tanshinone IIA. (B) Schematic representation of AOM/DSS-induced colitis-associated colorectal tumorigenesis modelling in mice.

TanshinoneIIA (Figure 1(A)) is a natural extract derived from Danshen (*Salviae miltiorrhizae* Bunge [Lamiaceae]), a Chinese medicinal herb identified by the Chinese Pharmacopoeia that is currently used to treat stroke, cardiovascular disorders, Alzheimer’s disease, diabetes, etc. with clinical efficacy (Gao et al. 2012; Robertson et al. 2014; Ji et al. 2017). Tanshinone IIA has exhibited wide-ranging effects such as its anti-angiogenic, antioxidative, anti-inflammatory, and antitumor activities (Shu et al. 2016). Regarding its antitumor roles, tanshinone IIA has been documented to have therapeutic effects in oesophageal, colorectal, lung, prostate, and gastric cancers by modulating cancer cell growth, apoptosis, invasion, migration, and drug resistance (Yang et al. 2005; Jieensinue et al. 2018; Gao et al. 2020). Tanshinone IIA may act as an adjuvant drug to inhibit the progression of human cancers in clinical practice (Zhang et al. 2019). To date, the effects and mechanisms of tanshinone IIA on colitis-associated CRC have not been fully investigated. In this study, we used a well-established azoxymethane (AOM)/dextran sulphate sodium (DSS)-induced murine CRC model to examine whether tanshinone IIA has a protective role and the mechanisms involved. We hypothesised that tanshinone IIA may protect against colitis-associated colorectal tumorigenesis through amelioration of intestinal inflammation in mice.

## Materials and methods

### Animals

Male C57BL/6 mice (aged 8–10 weeks) were purchased from the Laboratory Animal Centre of Tianjin Medical University (Tianjin, China). The mice were kept in standard housing cages under specific pathogen free conditions. All experimental procedures were reviewed and approved by the Tianjin Medical University Animal Care and Use Committee (approval No. ACUC-19-122) and were carried out in accordance with the NIH Guidelines for the Care and Use of Laboratory Animals.

### AOM/DSS-induced colorectal tumour mouse model

A murine colitis-associated colorectal tumour model was prepared as previously described (An et al. 2007; Li et al. 2019). The mice were randomly divided into three groups (*n* = 10): AOM/DSS treatment, AOM/DSS plus tanshinone IIA, and control. Briefly, the mice were given an intraperitoneal injection of AOM (10 mg/kg body weight; Sigma-Aldrich, USA) on the first day and then maintained on regular water and diet for one week. After that, the mice were fed with 2.5% DSS (40 kDa; Sigma-Aldrich) in their drinking water for one week and then received normal water for two weeks. This cycle was repeated three times. Tanshinone IIA (Sigma-Aldrich) was dissolved in DMSO and intraperitoneally administered to the mice (200 mg/kg body weight) every 2 days during induction of the CRC model (Figure 1(B)). Control mice were treated with vehicle or tanshinone IIA alone. Body weight was measured weekly. The mice were sacrificed at 12 weeks, and the colons were removed for further analysis. Each colon was cut open longitudinally and examined for tumour numbers and sizes. We obtained tumour volumes by measuring the length (*l*) and the width (*w*) and calculating the volume (*V = lw*^2^/2), as previously described (An et al. 2007). Parts of the colonic tissues were fixed in 10% neutral buffered formalin, processed, and embedded in paraffin. The sections (5 μm thick) were prepared and stained with haematoxylin and eosin using standard techniques. Fresh colonic tissue lysates were also collected for western blot or other analysis.

### Assay to detect intestinal integrity

We assayed intestinal integrity in mice as described previously (Liu et al. 2016). In brief, mice were given 200 μL of FITC-dextran (4 kDa, 500 mg/kg body weight, Sigma-Aldrich, USA) by gavage. After 4 h, serum samples were collected from the mice and assessed using a fluorimeter. Serial dilutions of FITC-dextran were used to generate a standard curve, and the serum concentrations of FITC-dextran in the mice were calculated accordingly.

### Measurement of colonic neutrophil infiltration and inflammation

To detect infiltrated neutrophils, colons were dissected from the mice and fixed in 4% paraformaldehyde at 4 °C overnight, followed by cryoprotection in 20% sucrose, and then embedded in a mixture of OCT compound and tissue freezing medium. The 8 μm cryosections were stained with rat anti-mouse Ly6G (2 μg/mL, Abcam) overnight at 4 °C and then incubated with FITC-conjugated donkey anti-rat IgG (1:200) for 1 h at room temperature. The sections were mounted with Vectashield mounting medium and examined with a fluorescence microscope.

We also measured the levels of colonic myeloperoxidase (MPO), reactive oxygen species (ROS), and multiple cytokines to characterise intestinal inflammation in AOM/DSS-treated mice. Briefly, colon tissues were dissected, rinsed with cold PBS, and cut into small pieces. The samples were homogenised in 50 mM phosphate buffer, and centrifuged at 10,000 *g* for 20 min at 4 °C to obtain the supernatant. The MPO and ROS levels were assayed using commercially available kits, according to the manufacturer’s protocols. In addition, we quantified a panel of multiple cytokines (IL-1β, IL-6, IL-10, IL-17A, IFN-γ, and TNF-α) in the colonic homogenates using the ProcartaPlex^TM^ Multiplex Immunoassay (Luminex) on a Bioplex-200 system with Bioplex Manger 5.0 software, according to the manufacturer’s protocol.

### Western blotting

The colonic tissues of mice were lysed in RIPA lysis buffer. After centrifugation at 10,000 *g* for 20 min at 4 °C, the supernatants were collected, and protein concentrations were measured using a BCA assay kit (Thermo Fisher, MA, USA). Electrophoresed samples were separated by SDS-PAGE and transferred to PVDF membranes (Millipore, MA, USA) and blocked using 5% fat-free milk for 1 h at room temperature. After being washed in PBST, the membranes were incubated with primary antibodies at 4 °C overnight. Then, the membranes were washed three times in PBST before the addition of HRP-labeled secondary antibodies. The signals were detected by the application of chemiluminescent HRP substrate (Millipore) on a Bio-Rad imaging system (Bio-Rad ChemiDoc MP). The primary antibodies against the following (Cell Signalling) were used: NF-κB p65, phospho-NF-κB p65, IκBα, phospho-IκBα, and β-actin.

### Assay for *in vitro* neutrophil activation

We collected peripheral blood from the mice in heparin-coated tubes by cardiac puncture and pooled the peripheral blood. After the lysis of red blood cells, we isolated neutrophils using the Ficoll gradient centrifugation method. Neutrophil purity was assessed using Wright-Giemsa staining and shown to be greater than 95% (Liu et al. 2016). We stimulated neutrophil activation by the addition of LPS (100 ng/mL) in DMEM with 10% FBS for 3 h. Tanshinone IIA was dissolved in DMSO and added to neutrophils with serial concentrations (10, 20, 40 µM). After treatment, the cells were harvested and homogenised in a lysis buffer. Then the homogenates were centrifuged at 10,000×*g* for 15 min at 4 °C, and the supernatants were analysed for MPO, ROS, and multiple cytokines (IL-1β, IL-6, IL-10, IL-17A, IFN-γ and TNF-α) as described above.

### Statistical analysis

All data were expressed as the mean ± SD. Student’s *t*-test (unpaired, 2-tailed) was used to analyse the statistical significance of differences between groups. The results were considered statistically significant when *p* < 0.05. All statistical analyses were calculated using GraphPad Prism.

## Results

### Tanshinone IIA alleviated AOM/DSS-induced colorectal tumorigenesis in mice

**Figure 2.**
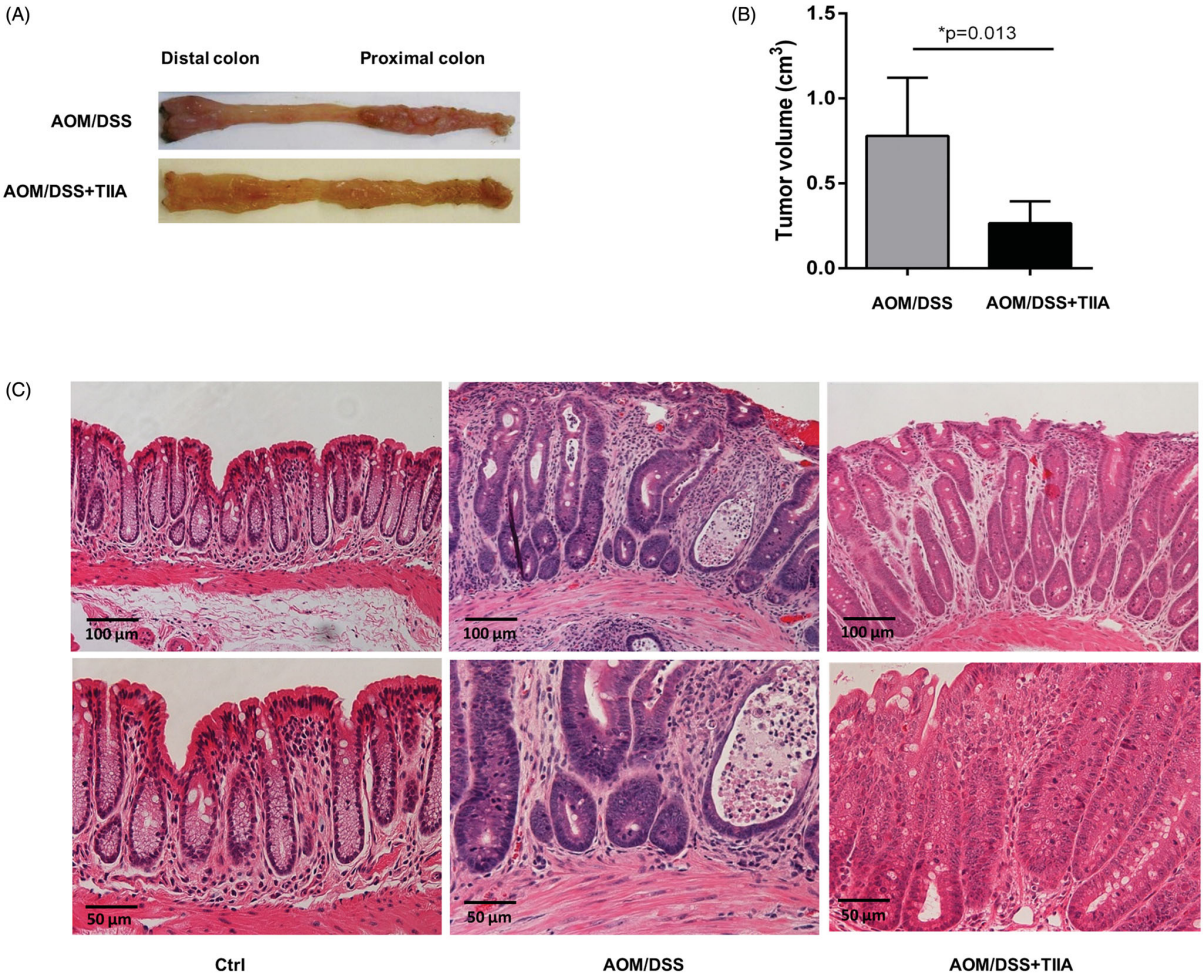
Inhibitory effects of tanshinone IIA on AOM/DSS-induced colorectal tumorigenesis in mice. (A) Representative luminal views of the colons from AOM/DSS-treated and AOM/DSS + tanshinone IIA-treated mice. (B) Quantification of colonic tumour volume in AOM/DSS-treated and AOM/DSS + tanshinone IIA-treated mice. Error bars represent the mean ± SD (*n* = 6 for each group). *p < 0.05 indicates a significant difference between mice. (C) Representative histopathological images of colon from control mice, mice treated with AOM/DSS or AOM/DSS plus tanshinone IIA. Tanshinone IIA significantly inhibited colorectal tumour formation and inflammatory cell infiltration in AOM/DSS-treated mice.

In this study, we used a well-established AOM/DSS murine model that mimics chronic intestinal inflammation to investigate whether tanshinone IIA has a therapeutic role in colitis-associated colorectal tumorigenesis. We observed that in AOM/DSS-treated mice, the colon and rectum developed multiple tumours at the experimental endpoint. Histological examination revealed obvious crypt destruction, architectural and cytological atypia, and massive inflammatory cell infiltration, as compared to the control mice receiving vehicle or tanshinone IIA alone, which did not exhibit any aberrant features. Most tumours exhibited extensive high-grade dysplasia or intramucosal carcinoma. In contrast, cotreatment with tanshinone IIA markedly reduced tumour volumes and caused a dramatic improvement in crypt structure and tumour formation in AOM/DSS-treated mice; most tumours exhibited low-grade dysplasia and much less infiltration of inflammatory cells (Figure 2). These data suggested that tanshinone IIA could significantly ameliorate colitis-associated colorectal tumorigenesis in mice.

### Tanshinone IIA ameliorated intestinal permeability and neutrophil infiltration in AOM/DSS-treated mice

**Figure 3.**
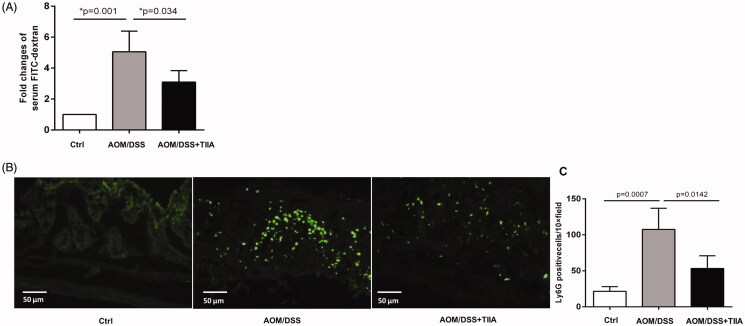
Tanshinone IIA improved intestinal permeability and reduced intestinal neutrophil infiltration. (A) Serum concentrations of FITC-dextran in AOM/DSS- and AOM/DSS + tanshinone IIA-treated mice were measured to assess intestinal permeability. Error bars represent the mean ± SD (*n* = 10 for each group). (B) Ly6G-positive neutrophil infiltrates in colonic sections from control mice, AOM/DSS- and AOM/DSS + tanshinone IIA-treated mice. (C) Positive staining areas per high-powered microscopic field (10×) were quantified based on six sections from three independent mice from each group. Error bars indicate the mean ± SD.

Next, we sought to explore the mechanisms by which tanshinone IIA protects against colorectal tumorigenesis. Intestinal permeability is frequently impaired during the course of colitis-associated colorectal cancer, which may in turn provoke inflammatory cell infiltration and enhance intestinal inflammation in a vicious cycle (Williams et al. 2015; Yu 2018; Becker et al. 2019). We used *in vivo* investigations to test whether tanshinone IIA may influence intestinal permeability. We treated the mice with FITC-dextran by gavage and measured serum concentrations of FITC-dextran to assess intestinal permeability. The results showed that serum concentrations of FITC-dextran in AOM/DSS-treated mice were drastically elevated than in the control mice, whereas cotreatment with tanshinone IIA caused a remarkable decrease in serum concentrations of FITC-dextran (Figure 3(A)), indicating that tanshinone IIA may improve intestinal permeability.

Impaired intestinal permeability leads to the infiltration of immune cells; of which, infiltrated neutrophils are an important feature in colitis-associated colorectal cancer (Hull et al. 2006; Becker et al. 2019; Lin et al. 2020). We therefore probed colonic tissues with anti-Ly6G, a specific antibody that recognises neutrophils, to characterise neutrophil infiltrates in the mucosa of AOM/DSS-treated mice. Compared to the control mice, AOM/DSS-treated mice showed a dramatic increase in Ly6G-positive cells around the mucosal area of, indicative of prominent neutrophil infiltration. In contrast, tanshinone IIA treatment significantly reduced Ly6G-positive cells in the mucosal tissues of AOM/DSS-treated mice (Figure 3(B,C)).

### Tanshinone IIA attenuated intestinal inflammation in AOM/DSS-treated mice

**Figure 4.**
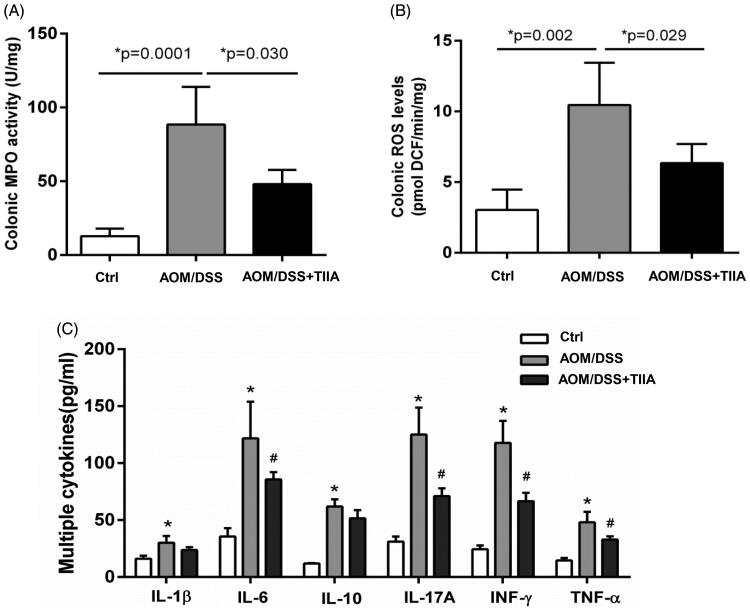
Tanshinone IIA inhibited intestinal inflammation in AOM/DSS-treated mice. (A) Colonic MPO activity (B) Colonic ROS levels were significantly elevated in AOM/DSS-treated mice, whereas tanshinone IIA markedly reduced their levels (mean ± SD, *n* = 6 for each group). (C) Multiple inflammatory cytokines were increased in the colonic tissues of AOM/DSS-treated mice when compared with their levels in the controls (*p < 0.05). Tanshinone IIA treatment decreased most of the detected cytokines (IL-6, IL-17A, INF-γ, TNF-α) in mice treated with AOM/DSS (^#^*p* < 0.05).

Increased neutrophil infiltration may elicit intestinal inflammatory responses, which further contribute to colorectal tumorigenesis (Terzić et al. 2010; Park and Kim 2018). To address this question, we measured MPO, ROS, and various cytokines in the colonic tissues of AOM/DSS-treated mice to assess the extent of intestinal inflammation. The results showed that the levels of MPO, ROS, and the inflammatory cytokines (IL-1β, IL-6, IL-10, IL-17A, IFN-γ and TNF-α) were all elevated in the colonic tissues of AOM/DSS-treated mice compared with the controls, indicating the occurrence of obvious intestinal inflammation. In contrast, tanshinone IIA cotreatment exhibited a suppressive effect on these indices (Figure 4).

**Figure 5.**
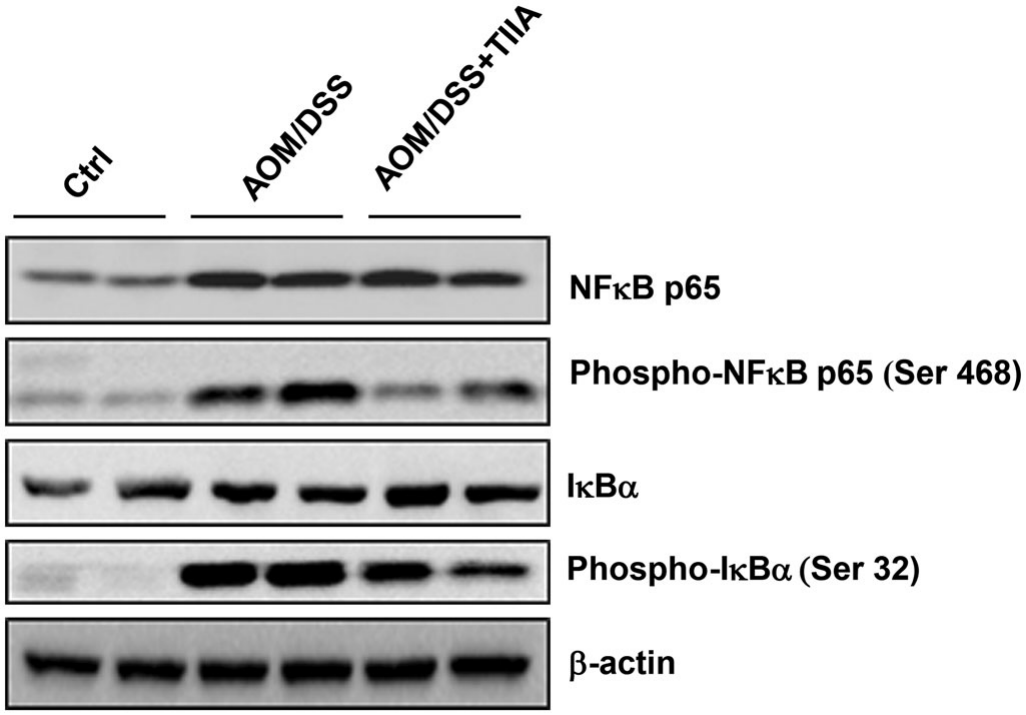
Tanshinone IIA downruglated the NF-κB signalling pathway. AOM/DSS treatment promoted the phosphorylation of p65-NF-κB and IκBα in colonic tissues of the mice, whereas administration of tanshinone IIA significantly inhibited activation of the NF-κB signalling pathway. β-Actin was used as a loading control. Data are representative of at least three experiments.

We further investigated the molecular mechanisms underlying the anti-inflammatory effects of tanshinone IIA. The NF-κB signalling pathway is well recognised to play a critical role in inflammatory diseases and tumorigenesis (Tago et al. 2019). Persistent activation of the NF-κB pathway can promote the malignant transformation and proliferation of colonic epithelial cells (Soleimani et al. 2020). We observed that in AOM/DSS-treated mice, the levels of NF-κB signalling components (p65-NF-κB, phosphorylated p65-NF-κB, phosphorylated IκBα) were upregulated compared with those in the control group, indicating that AOM/DSS promoted the activation of NF-κB signalling. Notably, tanshinone IIA cotreatment substantially decreased activation of the NF-κB signalling pathway, as evidenced by the reduced expression levels of both phosphorylated p65-NF-κB and IκBα (Figure 5). Taken together, these data suggested that tanshinone IIA may resolve intestinal inflammation by suppression of NF-κB pathway.

### Tanshinone IIA had an inhibitory effect on neutrophil activation *in vitro*

**Figure 6.**
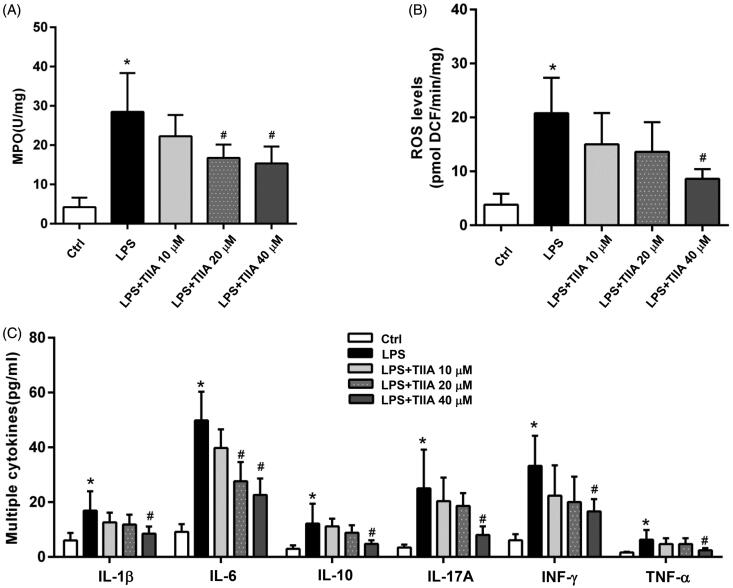
Tanshinone IIA suppressed neutrophil activation *in vitro*. (A) Isolated murine neutrophils showed a significant increase in MPO activity when treated with LPS, whereas coincubation with tanshinone IIA markedly inhibited MPO activities. (B) LPS-stimulated neutrophils produced high levels of ROS, whereas coincubation with tanshinone IIA markedly decreased ROS levels. (C) LPS-stimulated neutrophils produced high levels of cytokines (IL-1β, IL-6, IL-10, IL-17A, IFN-γ and TNF-α, all *p < 0.05 vs. the control cells), whereas coincubation with tanshinone IIA markedly decreased the levels of various cytokines in a dose-dependent manner (^#^*p* < 0.05 vs. LPS-treated cells). Experiments were repeated at least three times. Error bars represent the mean ± SD.

To validate these *in vivo* investigations, we isolated mouse neutrophils and treated them with LPS for 3 h in the absence or presence of tanshinone IIA with different concentrations (10, 20, 40 µM). LPS treatment significantly increased the activation of neutrophils, as evidenced by increased levels of MPO, ROS, and multiple cytokines in the supernatant of cultured cells, whereas tanshinone IIA coincubation markedly decreased these markers in a dose-dependent manner (Figure 6). These data suggested that tanshinone IIA has an inhibitory effect on neutrophil activation, which may account for its protection against colitis-associated colorectal tumorigenesis in mice.

## Discussion

Chronic intestinal inflammation is increasingly recognised due to its implication in the pathogenesis of CRC, which may induce the neoplastic transformation of colonic epithelial cells, promoting cellular proliferation and invasiveness, leading to the infiltration of immune cells and soluble mediators, which eventually provides a favourable microenvironment for tumour initiation and development (Park and Kim 2018; Pejin et al. 2017). Accumulating evidence has revealed that patients with inflammatory bowel disease (IBD) are at a high risk of developing CRC (Malhotra et al. 2018; Zhou et al. 2019). CRC patients are mainly treated with surgery supplemented with other therapeutic options, such as chemotherapy and radiotherapy (Johnson et al. 2013; Ganesh et al. 2019). The resolution of intestinal inflammation has been suggested as an important therapeutic strategy to prevent and treat CRC (Pejin et al. 2013; Zhao et al. 2020). To date, interest in exploring potential agents with anti-inflammatory properties for the prevention and treatment of CRC has been increasing.

Previous studies have reported that tanshinone IIA, a natural product isolated from a Chinese medicinal herb, displayed notable protective effects against a variety of inflammatory conditions including colitis (Yang et al. 2005; Liu et al. 2016). In addition, tanshinone IIA has also been frequently used as an adjunct drug to retard the progression of many cancers (Dong et al. 2011). The tumour-suppressive effects of tanshinone IIA are thought to be attributed to its influence on cell growth, migration and invasion, and enhancement of cell apoptosis (Xing et al. 2015; Gao et al. 2020). In this study, we used an AOM/DSS-induced colitis-associated colorectal tumour model, which resembles human CRC, to evaluate whether tanshinone IIA has beneficial effects on colorectal tumorigenesis, and thereby may have potentials as a therapy in clinical practice. Our data showed that mice treated with AOM/DSS developed typical tumours in the colon and rectum; most of the epithelia exhibited moderate or severe dysplasia, irregular crypt arrangement, and inflammatory cell infiltration of the mucosa. Notably, tanshinone IIA significantly reduced the severity of glandular hyperplasia and the extent of inflammatory cell infiltration. These results suggest that tanshinone IIA has a therapeutic effect on colitis-associated colorectal tumorigenesis, thereby suggesting its consideration as a promising agent for the prevention and treatment of CRC.

We next sought to delineate the mechanisms by which tanshinone IIA confers its protection against colorectal tumorigenesis in this model. Indeed, tanshinone IIA has been shown to be protective against a variety of inflammatory disease models, including DSS-induced colitis in mice (Zhang et al. 2015; Liu et al. 2016). DSS is a heparin-like polysaccharide that can impair epithelial integrity, thereby increasing intestinal permeability, which may further lead to immune cell infiltration and subsequent intestinal inflammation (Liu et al. 2016). Our data demonstrated robust inflammation in the colonic tissues of AOM/DSS-treated mice, as evidenced by damaged intestinal permeability, the prominent infiltration of neutrophils, a significant increase in the release of MPO and ROS and high levels of inflammatory cytokines, which may be involved in the initiation and development of colorectal tumours. Administration of tanshinone IIA significantly ameliorated intestinal inflammation in AOM/DSS-treated mice.

Furthermore, a remarkable improvement in intestinal permeability was observed, accompanied by reduced neutrophil infiltration and the decreased production of MPO, ROS and multiple inflammatory cytokines, which may largely account for the protective effects of tanshinone IIA in this model. The NF-κB signalling pathway plays a key role in colitis-associated colorectal tumorigenesis. It has been reported that activation of the NF-κB signalling pathway may be essential for the progression of colitis to cancer (Wan et al. 2019; Jiang et al. 2020).

Our results showed that AOM/DSS-treated mice exhibited elevated levels of NF-κB signalling components, consistent with the observed intestinal inflammation. Tanshinone IIA inhibited these increases, suggesting that it may exert anti-inflammatory effects through the inhibition of NF-κB signalling pathway.

In addition, as mentioned before, infiltrated neutrophils resulting from impaired intestinal integrity may lead to accelerated intestinal inflammation. We questioned whether tanshinone IIA exerts its anti-inflammatory effects by targeting neutrophils. We isolated and treated murine neutrophils with LPS in the presence or absence of tanshinone IIA. We found that tanshinone IIA could inhibit LPS-stimulated neutrophil activation, as demonstrated by the decreased production of MPO, ROS and inflammatory cytokines, which was consistent with the *in vivo* findings.

In summary, our data suggest that tanshinone IIA can be used for the prevention and treatment of colitis-associated colorectal tumorigenesis, which is likely due to its bioactivity in ameliorating intestinal inflammation by inhibition of neutrophil activation.
